# Activation of an NLRP3 Inflammasome Restricts *Mycobacterium kansasii* Infection

**DOI:** 10.1371/journal.pone.0036292

**Published:** 2012-04-30

**Authors:** Chang-Chieh Chen, Sheng-Hui Tsai, Chia-Chen Lu, Shiau-Ting Hu, Ting-Shu Wu, Tsung-Teng Huang, Najwane Saïd-Sadier, David M. Ojcius, Hsin-Chih Lai

**Affiliations:** 1 Green Energy and Environment Research Laboratories, Industrial Technology Research Institute, Chutung, Hsinchu, Taiwan, Republic of China; 2 Institute of Microbiology and Immunology, School of Life Science, National Yang-Ming University, Taipei, Taiwan, Republic of China; 3 Department of Respiratory Therapy, Fu Jen Catholic University, Taipei, Taiwan, Republic of China; 4 Department of Microbiology and Immunology, School of Medicine, National Yang-Ming University, Taipei, Taiwan, Republic of China; 5 Department of Internal Medicine, Chang Gung Memorial Hospital and Graduate Institute of Clinical Medical Sciences, Chang Gung University, Kweishan, Taoyuan, Taiwan, Republic of China; 6 Center for Molecular and Clinical Immunology, Chang Gung University, Kweishan, Taoyuan, Taiwan, Republic of China; 7 Department of Medical Biotechnology and Laboratory Sciences, Chang Gung University, Kweishan, Taoyuan, Taiwan, Republic of China; 8 Laboratory of Nanomaterials, Chang Gung University, Kweishan, Taoyuan, Taiwan, Republic of China; 9 Research Center of Bacterial Pathogenesis, Chang Gung University, Kweishan, Taoyuan, Taiwan, Republic of China; 10 Health Sciences Research Institute and School of Natural Sciences, University of California Merced, Merced, California, United States of America; University of Maryland, United States of America

## Abstract

*Mycobacterium kansasii* has emerged as an important nontuberculous mycobacterium pathogen, whose incidence and prevalence have been increasing in the last decade. *M. kansasii* can cause pulmonary tuberculosis clinically and radiographically indistinguishable from that caused by *Mycobacterium tuberculosis* infection. Unlike the widely-studied *M. tuberculosis*, little is known about the innate immune response against *M. kansasii* infection. Although inflammasome activation plays an important role in host defense against bacterial infection, its role against atypical mycobacteria remains poorly understood. In this report, the role of inflammasome activity in THP-1 macrophages against *M. kansasii* infection was studied. Results indicated that viable, but not heat-killed, *M. kansasii* induced caspase-1-dependent IL-1β secretion in macrophages. The underlying mechanism was found to be through activation of an inflammasome containing the NLR (Nod-like receptor) family member NLRP3 and the adaptor protein ASC (apoptosis-associated speck-like protein containing a CARD). Further, potassium efflux, lysosomal acidification, ROS production and cathepsin B release played a role in *M. kansasii*-induced inflammasome activation. Finally, the secreted IL-1β derived from caspase-1 activation was shown to restrict intracellular *M. kansasii*. These findings demonstrate a biological role for the NLRP3 inflammasome in host defense against *M. kansasii*.

## Introduction

*Mycobacterium kansasii* is an acid-fast bacillus that has emerged as an important pathogen of the group of nontuberculous mycobacteria (NTM). It is the second most-common nontuberculous opportunistic mycobacterial infection linked with AIDS, surpassed only by *Mycobacterium avium* complex (MAC) [1]–[3]. Furthermore, *M. kansasii* infects both immunocompetent and immunocompromised patients [4]–[9].

Although geographical variability of infection exists, *M. kansasii* is the most common cause of NTM-induced lung diseases in the United Kingdom and Western Europe [10]–[14]. *M. kansasii* causes pulmonary infection that resembles tuberculosis clinically and radiographically and is indistinguishable from *Mycobacterium tuberculosis* infection [12], [13], [15]. Comorbidity diseases are frequently closely related with *M. kansasii* pulmonary infection, including chronic obstructive pulmonary disease, bronchiectasis, pneumonoconiosis, previous tuberculosis, or carcinoma [16], [17]. In addition, extrapulmonary infection of *M. kansasii* can cause gastroenteritis, lymphadenitis, osteomyelitis, synovitis, cellulitis, empyema or pericarditis [14], [18], [19]. Furthermore, disseminated *M. kansasii* infections also commonly occur, especially in immunocompromised patients with advanced AIDS [20], [21]. Comparatively, unlike the widely-studied *M. tuberculosis*, most reports on *M. kansasii* focus on epidemiological and clinical features of infection [22]–[24]. Little is known about the innate immune response against *M. kansasii* infection.

Macrophages represent the first line of host defense against most bacterial pathogens. Following interaction with the bacteria, macrophages initiate inflammatory responses by secreting cytokines and chemokines [25], [26]. Among these, one of the key proinflammatory cytokines for antimicrobial responses is interleukin-1β (IL-1β) [27]. Two signaling systems control the synthesis, processing and secretion of IL-1β. Pathogen-recognition receptors such as Toll-like receptors (TLRs) control synthesis of pro-IL-1β, and the nucleotide binding and oligomerization domain (NOD)-like receptors (NLRs) lead to inflammasome activation and IL-1β maturation and secretion [28].

During infection with pathogenic bacteria, assembly and activation of the inflammasome result in caspase-1 activation and IL-1β secretion, which are critical for an effective immune response [29]. To date, among the four major inflammasomes described [30], the most thoroughly characterized is the NLRP3 inflammasome, which is activated by a number of diverse stimuli, including whole pathogens, microbial components and danger signals [31]. Upon activation, NLRP3 oligomerizes and recruits the adaptor protein ASC (apoptosis-associated speck-like protein containing a CARD) through pyrin domain interactions. In turn, procaspase-1 is recruited by ASC via CARD-CARD interactions, thus forming the NLRP3 inflammasome and leading to caspase-1 activation. Caspase-1 is synthesized as a 45 kDa precursor (p45) before being cleaved into 20 kDa (p20) and 10 kDa (p10) mature proteins that form a hetero-tetrameric complex that express the enzymatic activity [32]. Thus, the appearance of p20 and p10 in culture supernatants reflects caspase-1 activation [33]. Similarly, upon stimulation of a pathogen recognition receptor such as the TLRs, proinflammatory cytokine IL-1β is generated as a 31 kDa proform which is proteolytically processed to the biologically active 17 kDa form by caspase-1 [34], and then released into the extracellular space through mechanisms that remain poorly characterized [35], [36].

Previous studies have indicated that *M. marinum* and *M. tuberculosis* and their components can activate an inflammasome consisting of NLRP3 and ASC [37], [38]. In addition to live *M. tuberculosis*, the ESAT-6 protein from the mycobacteria has been shown to induce activation of NLRP3/ASC inflammasome maturation and release of IL-1β from THP-1 macrophages [39]. Furthermore, studies using primary macrophages demonstrated that *M. marinum* activates the NLRP3/ASC inflammasome in an ESX-1-dependent manner [40]. Recently, a rapidly growing NTM, *M. abscessus*, has been reported to activate the NLRP3/ASC inflammasome via dectin-1/Syk-dependent signaling and the cytoplasmic scaffold protein p62/SQSTM1 in human macrophages [41]. However, whether *M. kansasii*, a slowly growing NTM, infection could induce caspase-1 activation and IL-1β secretion via the inflammasome activation has not been reported yet. Therefore, the role of inflammasome activation and secretion of IL-1β in prevention of *M. kansasii* infection was addressed in this study. Using the human macrophage cell line THP-1, we demonstrated that live intracellular *M. kansasii* triggers the activation of the NLRP3/ASC complex, caspase-1 activation, and IL-1β secretion. We further showed that potassium efflux, lysosomal acidification, cathepsin B release and production of reactive oxygen species (ROS) are required for the activation of the inflammasome. Finally, we demonstrate a major role for the secreted IL-1β in controlling *M. kansasii* infection. These results demonstrate an important biological function for the NLRP3 inflammasome in host defense against *M. kansasii* infection.

## Results

### Live intracellular *M. kansasii* triggers caspase-1 activation and IL-1β secretion in macrophages

**Figure 1:**
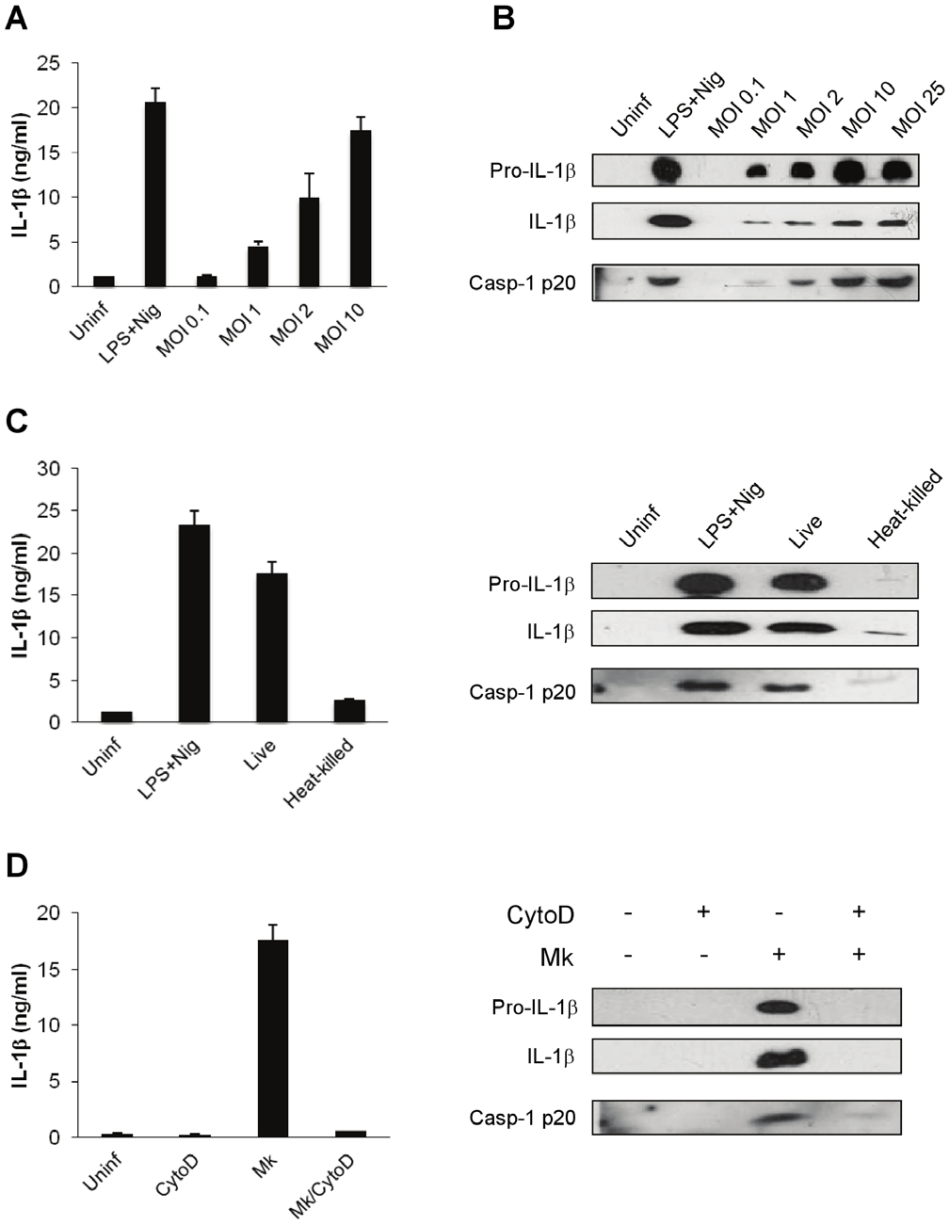
Live intracellular *M. kansasii* activates caspase-1 and induces IL-1β secretion in THP-1 derived macrophages. Macrophages were infected by *M. kansasii*, followed by analysis of caspase-1 activation and IL-1βsecretion in culture media. (**A**) The amount of IL-1β secreted from infected macrophages at an MOI of 0.1–10 for 16 h. **(B**) The secreted mature caspase-1 and IL-1β from infected macrophages at an MOI of 0.1–25 for 16 h. (**C**) Macrophages were challenged with live or heat-killed *M. kansasii* at an MOI of 10 for 16 h. Secreted IL-1β was measured by ELISA (left panel). Caspase-1 p20 and 17-kDa IL-1β were analyzed by immunoblotting (right panel). (**D**) Macrophages were treated with 1 µg/ml CytoD for 60 min to block phagocytosis and then incubated with *M. kansasii* at an MOI of 10 in the presence or absence of CytoD. Secreted IL-1β was measured by ELISA (left panel); activated caspase-1 and mature IL-1β were detected by Western blot analysis (right panel). Cells treated with 1 µg/ml LPS for 3 h and 1 µM nigericin for 1.5 h were used as control (LPS+Nig). Values represent the mean ± standard deviations of at least three independent experiments.

**Figure 2:**
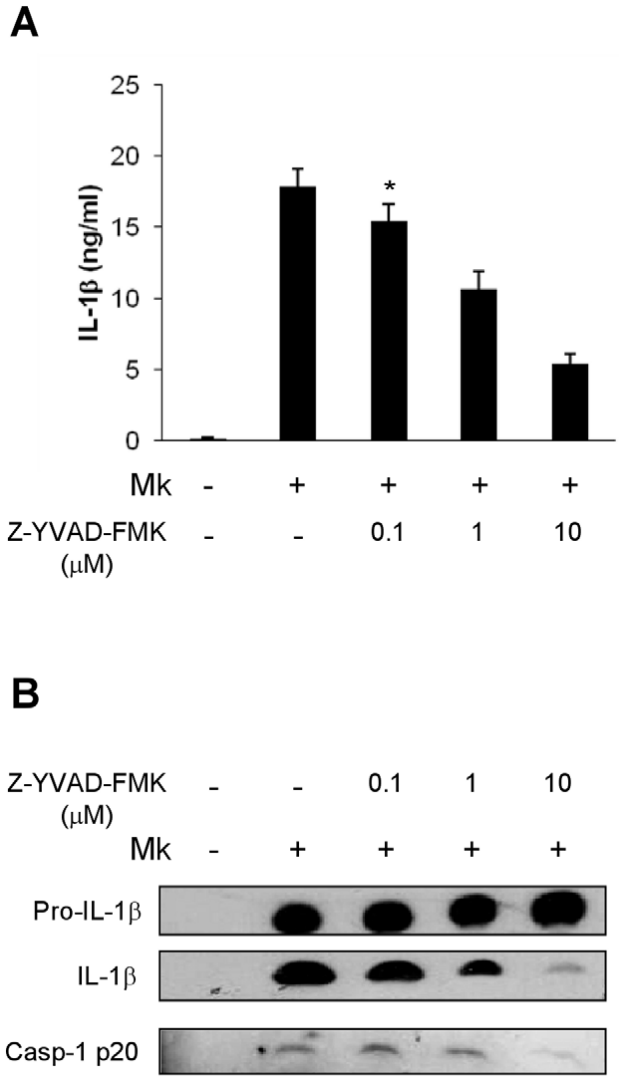
Release of mature IL-1β by *M. kansasii*-infected cells requires caspase-1 activation. Macrophages were infected with *M. kansasii* in the presence or absence of the caspase-1 inhibitor, Z-YVAD-FMK. At 16 h post-infection, secreted IL-1βwas quantified by ELISA (**A**), and secreted caspase-1 p20 and mature IL-1β were analyzed by Western blot analysis (**B**). Error bars represent standard deviations from at least three independent experiments. ** denote a *p* value of <0.001 compared to untreated infected cells.

To determine whether *M. kansasii* infection could induce caspase-1 activation and IL-1β secretion, THP-1 macrophages were challenged with *M. kansasii* at various multiplicities of infection (MOI) for 16 h. Caspase-1 cleavage and IL-1β processing were analyzed by ELISA and immunoblotting. Compared to untreated cells, bacterial challenge resulted in a dose-dependent caspase-1 activation and IL-1β secretion (Figure 1A and B), indicating that *M. kansasii* infection activates caspase-1 and promotes IL-1β release. By contrast, heat-killed *M. kansasii* failed to induce caspase-1 activation nor IL-1β secretion (Figure 1C). As macrophages can engulf mycobacteria, whether internalization of *M. kansasii* is required for the processing of IL-1β and caspase-1 was examined. Cytochalasin D, an inhibitor of actin polymerization, was used to block phagocytosis before *M. kansasii* infection. As shown in Figure 1D, caspase-1 activation and IL-1β maturation were abolished when internalization of *M. kansasii* by macrophages was inhibited. To determine whether activated caspase-1 is responsible for *M. kansasii* induced maturation and secretion of IL-1β, macrophages were pretreated with Z-YVAD-FMK, a cell-permeable and irreversible caspase-1 inhibitor. When caspase-1 activity was inhibited, the release of mature IL-1β into supernatants was reduced in a dose dependent manner, and both IL-1β processing and caspase-1 activation were reduced (Figure 2). Thus, viable intracellular *M. kansasii* induce caspase-1-dependent IL-1β secretion from macrophages.

### The NLRP3/ASC inflammasome contributes to caspase-1 activation and IL-1β secretion during *M. kansasii* infection

**Figure 3:**
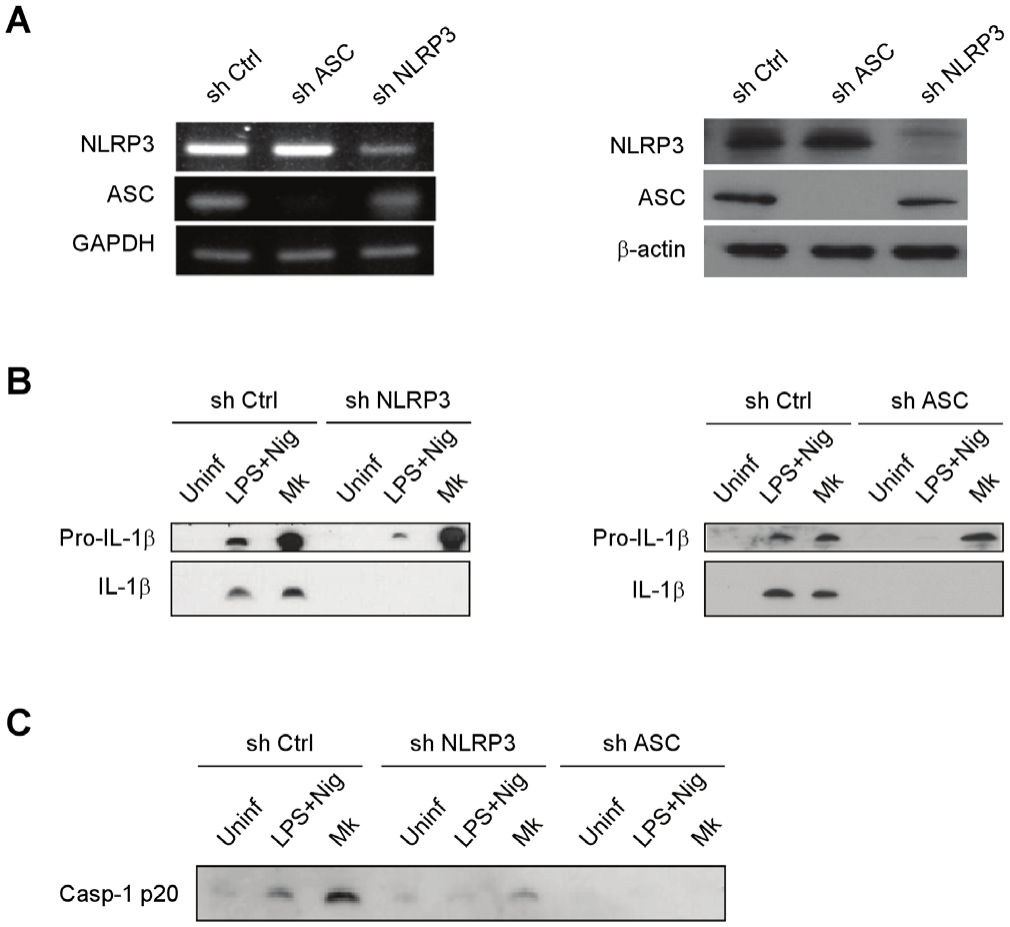
*M. kansasii* induces caspase-1 activation and IL-1β secretion through the NLRP3/ASC-dependent pathway. (**A**) THP-1 cells were stably transfected with shRNAs that target NLRP3 or ASC, and mRNA expression of NLRP3 and ASC was determined by RT-PCR and compared with nontarget control (sh Ctrl) (left panel). Protein levels of NLRP3 or ASC in the respective knockdown cells were analyzed by Western blot analysis (right panel). (**B** and **C**) NLRP3, ASC, or nontarget control (sh Ctrl) knockdown cells were infected with *M. kansasii* at an MOI of 10 for 16 h. Secreted IL-1β(**B**) and activated caspase-1 (**C**) were examined by Western blot analysis.

To define the role of NLRP3 inflammasome components in activation of caspase-1 by *M. kansasii* infection, NLRP3 or ASC in THP-1 cells were depleted by shRNA. Compared to non-target shRNA control, mRNA and protein levels of NLRP3 or ASC in the respective knockdown cells were significantly reduced (Figure 3A). NLRP3, ASC, or nontarget control knockdown THP-1 cells were then challenged with *M. kansasii* at an MOI of 10 for 16 h, and caspase-1 activation was revealed by the appearance of caspase-1 p20 and IL-1β p17 in Western blots. When compared to non-target control cells, depletion of either NLRP3 or ASC caused a significant reduction in the amount of secreted IL-1β(Figure 3B), while IL-6 production was unimpaired (Figure S1). Concomitantly, significant reduction of caspase-1 p20 levels was also observed (Figure 3C). Taken together, the results show that NLRP3 and ASC are required for *M. kansasii* induced caspase-1 activation and IL-1β processing in macrophages.

### Potassium efflux, lysosomal acidification, ROS production and cathepsin B release are involved in *M. kansasii*-induced inflammasome activation

**Figure 4:**
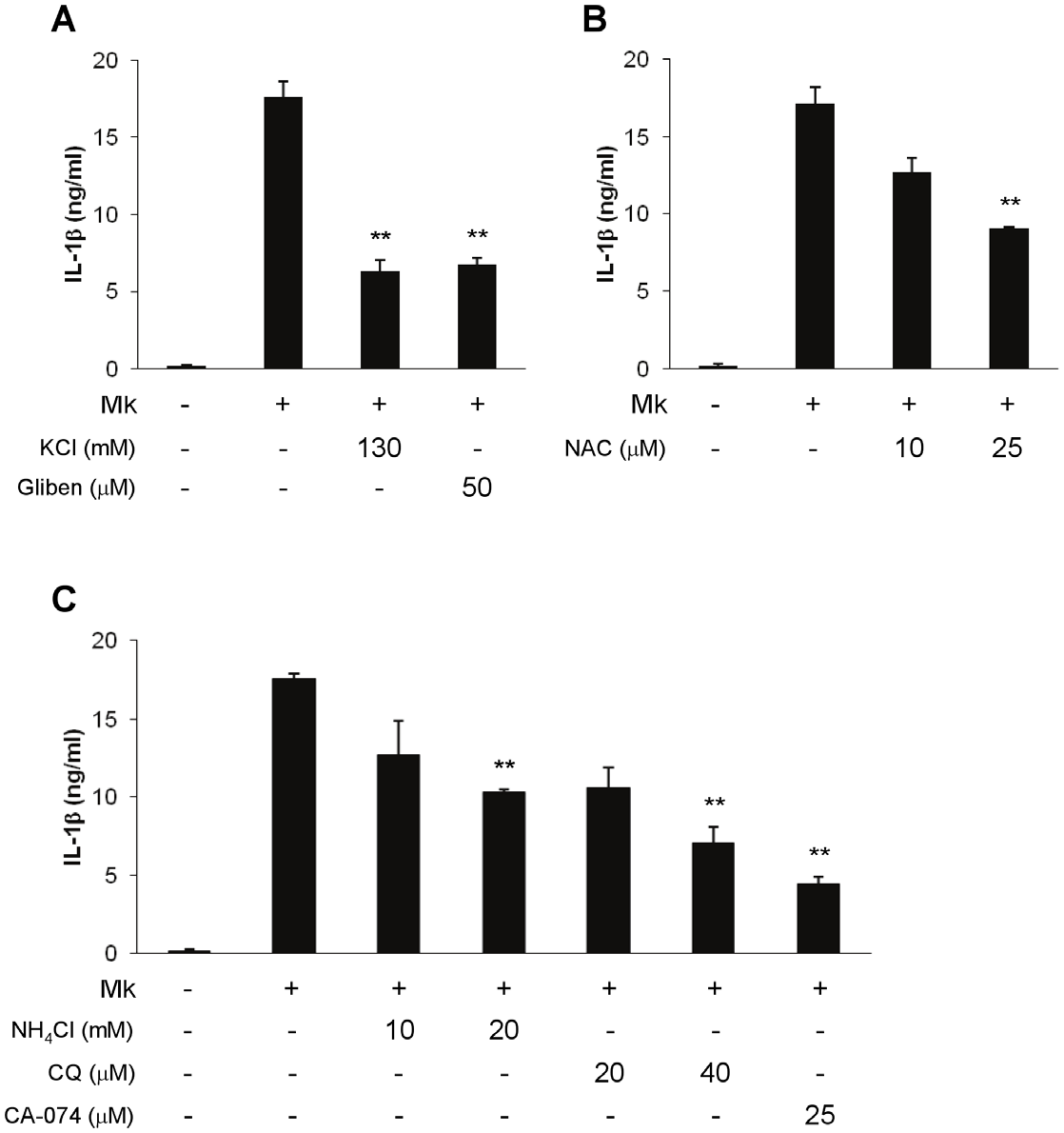
Potassium efflux, lysosomal acidification, cathepsin B release and ROS production are involved in inflammasome activation by *M. kansasii*. (**A**) Macrophages were treated with KCl and Glibenclamide (Gliben) at the indicated concentration for 30 min, and subsequently infected with *M. kansasii* at an MOI of 10 for 16 h. Supernatants from infected cells were harvested and assayed for IL-1β by ELISA. (**B** and **C**) THP-1 derived macrophages were infected with *M. kansasii* at an MOI of 10. N-acetyl cysteine (NAC), NH_4_Cl, chloroquine (CQ) and CA-074-Me (CA-074) were added at the indicated concentration as described in Materials and Methods. Supernatants harvested at 16 h post-infection were assayed for IL-1βsecretion by ELISA. Error bars represent standard deviations of at least three independent experiments, and significance was calculated using a two-tailed *t* test. ** denote a *p* value of <0.001 compared to untreated infected cells.

Previous studies have reported a number of signaling mechanisms playing important roles in activating the NLRP3/ASC inflammasome, including potassium efflux, lysosomal acidification, generation of reactive oxygen species (ROS) and cathepsin B release from lysosomes. To explore whether any of these factors are involved in *M. kansasii*-induced inflammasome activation, a series of defined inhibitors were used to treat *M. kansasii*-challenged THP-1 macrophages. The amount of secreted IL-1β in culture supernatants was used as an indicator of inflammasome activation. A high extracellular potassium concentration (130 mM) was first used to test if inhibiting K^+^ efflux can influence *M. kansasii*-mediated activation of the NLRP3/ASC inflammasome. Following infection of THP-1 macrophages in high extracellular [K^+^], *M. kansasii*-induced IL-1β release was significantly inhibited (Figure 4A). Moreover, glibenclamide, a selective inhibitor for ATP-dependent potassium channels, was used to block potassium efflux. Addition of 50 µM glibenclamide to macrophages prior to bacterial challenge significantly reduced IL-1β secretion (Figure 4A). Since ROS generation was reported to activate the NLRP3/ASC inflammasome [42], the anti-oxidant, N-acetyl cysteine (NAC), was used to determine whether ROS are involved in *M. kansasii*-mediated NLRP3/ASC inflammasome activation. IL-1β secretion was significantly reduced upon treatment with 25 µM NAC (Figure 4B), indicating that ROS also contributes to NLRP3/ASC inflammasome activation during *M. kansasii* infection. As the NLRP3 inflammasome can be activated due to lysosomal damage and release of cathepsin B, ammonium chloride (NH_4_Cl) and chloroquine diphosphate (CQ), which inhibit endosomal-lysosomal system acidification, and CA-074-Me, which acts as a cell-permeable inhibitor of thiol proteases, were used to block lysosomal acidification and cathepsin B activity, respectively. The three inhibitors resulted in a significant reduction in IL-1β release after *M. kansasii* challenge (Figure 4C), suggesting a role for lysosomes in NLRP3/ASC inflammasome activation in response to *M. kansasii* infection. However, these marked inhibitory effects were not due to cytotoxicity from these treatments (Fig S2). Thus, the results suggest that NLRP3/ASC activation during *M. kansasii* infection involves most of the pathways known to activate the NLRP3 inflammasome: potassium efflux, lysosomal acidification, cathepsin B release, and ROS production.

### IL-1β derived from caspase-1 activation restricts *M. kansasii* infection

**Figure 5:**
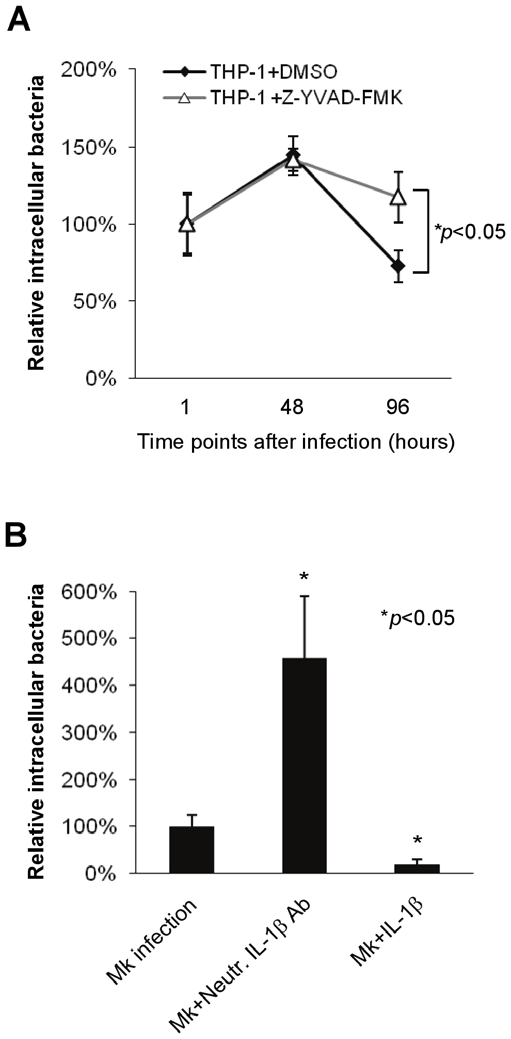
Caspase-1 activation and IL-1β secretion restrict *M. kansasii* growth. (**A**) THP-1 macrophages were infected with *M. kansasii* at an MOI of 1 for 1 h, before treatment with 50 µM caspase-1 inhibitor (Z-YVAD-FMK) or dimethylsulfoxide (DMSO) alone as control. Cells were then lysed and the intracellular bacterial load was quantified at indicated time points. (**B**) The *M. kansasii*-infected macrophages (MOI 1, 1 h) were treated with neutralizing antibodies specific for IL-1βor with exogenous IL-1β for 48 h. The intracellular bacterial CFU was then determined. Results represent the mean ± standard deviations of three independent experiments. Data were analyzed by Student's *t* test. *p<0.05 compared to untreated infected cells.

Caspase-1 activation has been reported to restrict bacterial infection either directly, by affecting bacterial growth, or indirectly, through IL-1β-mediated inhibition of infection [38], [39], [43]–[46]. To address the contribution of caspase-1 in the control of *M. kansasii* infection, macrophages were treated with caspase-1 inhibitor, Z-YVAD-FMK, before *M. kansasii* challenge. Intracellular bacterial growth was evaluated by quantifying the colony forming units (CFUs) for 96 h following bacterial infection. The number of CFUs recovered from macrophages treated with caspase-1 inhibitor was greater than those from untreated cells, indicating that caspase-1 activation contributes to control of *M. kansasii* infection in macrophages (Figure 5A).

Studies from knockout mice with deficiency in either IL-1β or its receptor, IL-1R, suggested that IL-1β plays an important role in the immune response to *M. tuberculosis* or *M. kansasii* infection in mice [47]–[49]. Whether IL-1β also affects *M. kansasii* infection of human macrophages *in vitro* was next investigated. THP-1 macrophages were treated with IL-1β neutralizing antibody during bacterial infection. The intracellular bacterial number was evaluated by quantifying the CFUs at 48 h post-infection. Blocking IL-1β signaling with IL-1β specific neutralizing antibody resulted in a nearly 5-fold increase in the number of intracellular *M. kansasii* (Figure 5B). Conversely, exogenously-added IL-1βsignificantly reduced the bacterial counts in macrophages. These results suggest that IL-1β secreted due to NLRP3 inflammasome activation is critical for the control of intracellular *M. kansasii*, and that caspase-1 most likely affects infection indirectly, through processing of IL-1β.

## Discussion

In this report, we demonstrated that live and intracellular *M. kansasii* can trigger caspase-1 activation and IL-1β secretion from human macrophages by activating the NLRP3/ASC inflammasome. Heat-killed and extracellular *M. kansasii* failed to activate the NLRP3/ASC inflammasome, suggesting that simple surface contact of *M. kansasii* with macrophages is not sufficient for activation of the inflammasome. Although the precise mechanisms behind responsible for NLRP3/ASC inflammasome activation are still under investigation, our results suggest that *M. kansasii* infection of macrophages can induce potassium efflux, cathepsin B release and ROS production, all of which are involved in NLRP3/ASC inflammasome activation. Additionally, the results from the intracellular survival assay revealed that *M. kansasii*-induced caspase-1 activation and subsequent IL-1β secretion restrict growth of intracellular *M. kansasii*. This is the first report showing inflammasome activation induced by *M. kansasii* and demonstrating a biological function for the NLRP3/ASC inflammasome in host defense against this slow-growing NTM.

*M. kansasii* can cause a pneumonia that resembles classical lung tuberculosis in many features, reflecting similarities in the pathogenesis between *M. kansasii* and *M. tuberculosis* infection; however, several reports have also referred to differences in host and cellular responses to *M. kansasii* and *M. tuberculosis*. During infection by *M. tuberculosis* or *M. avium* complex which is also a pathogenic NTM, CD4^+^ T cells, interferon (IFN)-γ, or IL-12p40 are crucial for the development of protective immunity in mice [50]–[57]. However, CD4^+^ and IFN-γ deficient mice display normal resistance against pulmonary infection with *M. kansasii*, indicating that a T helper cell type 1 response is not sufficient for control of *M. kansasii* infection [58]. Since macrophages are the first line of defense against pathogens such as mycobacteria, and following phagocytosis, *Mycobacterium* species reside and multiply in macrophages, we characterized the ability of macrophages to control *M. kansasii* infection *in vitro*. Recent studies have demonstrated that the pathogenic mycobacteria, *M. tuberculosis*, *M. marinum* and *M. abscessus*, induce activation of the NLRP3/ASC inflammasome and subsequent release of IL-1βin macrophages [37], [39]–[41], [59], [60]. Here, we found that macrophages infected by *M. kansasii* also secrete IL-1β via activation of the NLRP3 inflammasome, suggesting that all pathogenic mycobacteria may stimulate inflammasome activation and IL-1β secretion.

The mechanisms leading to NLRP3 inflammasome activation comprise mainly three signaling pathways, including potassium efflux, cathepsin B release, and generation of ROS [30]. The current results showed that ROS production, potassium efflux, lysosomal acidification, and cathepsin B release are all required for *M. kansasii* induced NLRP3-dependent caspase-1 activation and IL-1β secretion. In this study, a potent cathepsin B inhibitor, CA-074-Me, was used to determine the involvement of cathepsin B in NLRP3 inflammasome activation during *M. kansasii* infection. However, CA-074-Me has been proposed to act on other cellular proteases [61], suggesting the possibility that CA-074-Me may inhibit *M. kansasii*-induced IL-1β secretion through a cathepsin B-independent manner. Even so, a recent study with cathepsin B knockdown cells confirmed that cathepsin B is involved in inflammasome activation upon mycobacteria infection [62].

It has been shown that infection by *M. tuberculosis* and *M. marinum* may induce ESX-1-dependent NLRP3 inflammasome activation [37], [39], [40], [60]. ESX-1 has been identified as a critical virulence factor in pathogenic mycobacteria and is involved in immune signaling, cytolysis, phagosome escape and membrane pore formation [63]–[67]. As *M. kansasii* also expresses ESX-1 [68], it seems reasonable to assume that pore formation of cell membranes by *M. kansasii* ESX-1 could cause potassium efflux and subsequent activation of the NLRP3 inflammasome. In addition, ESX-1-induced vacuole escape could lead to lysosomal damage and cathepsin B release, which have been implicated as potential activators of the NLRP3 inflammasome [63], [66], [67]. Recently, ESAT-6, one of the secreted effectors of ESX-1, has been shown to be a potent activator of the NLRP3/ASC inflammasome, possibly due to its membrane-lysing activity [39]. Genetic analysis and the nucleotide sequences revealed that the virulence genes, *esx-1* and *esat-6* of *M. tuberculosis*, are lacking in most environmental mycobacteria except for *M. kansasii* and *M. marinum* [69]–[71], both of which can cause disease in apparently immunocompetent persons. Moreover, analysis of immunoblotting with specific antibodies indicated that the ESAT-6 was expressed by *M. kansasii* [68], [71]. Thus, we propose that the activation of the NLRP3 inflammasome by live intracellular *M. kansasii* might be through ESX-1 or ESAT-6.

Although the role of ROS in the activation of NLRP3 inflammasome is controversial [72]–[78], several studies have demonstrated that ROS are required for inflammasome activation during bacterial infections [42], [79]–[82]. In this study, NAC significantly diminished IL-1β secretion triggered by mycobacterial infection, suggesting that ROS are involved in *M. kansasii*-induced NLRP3 inflammasome activation; however the source of ROS is currently unknown. The intracellular ROS are mainly generated from two sources: the mitochondrial electron transport chain complex, and NADPH oxidase at the plasma membrane or phagosomal membrane of phagocytes [45], [83]–[85]. It has been shown that *M. tuberculosis* infection leads to intracellular ROS production via NADPH oxidase at phagosomal membranes, and ESAT-6 treatment induces a robust burst of intracellular ROS production in human alveolar epithelial cells [86], [87]. Moreover, a low intracellular K^+^ concentration has been proposed to trigger ROS generation [73], indicating the possibility that ESAT-6, a pore-forming protein, could initiate potassium efflux and subsequently ROS production. Recent studies indicated that besides NADPH oxidase, NLRX1, a member of the Nod-like receptor (NLR) family that is localized in mitochondria, can enhance ROS production following infections by *Shigella flexneri* and *Chlamydia trachomatis* infection [80], [88]. Moreover, mitochondrial dysfunction-derived ROS has been shown to activate NLRP3 inflammasome [89], [90]. Thus, whether ESAT-6, NADPH oxidase, NLRX1 or mitochondrial dysfunction are involved in *M. kansasii*-induced ROS production remains to be determined.

Caspase-1, known as an inflammatory caspase, plays a key role in the innate immune response of macrophages to various infections [91]–[93]. Activation of caspase-1 is responsible for the processing and secretion of the proinflammatory cytokines, IL-1β and IL-18 [34], [94], [95]. In addition, active caspase-1 mediates either cell death or survival, and regulates unconventional secretion of leaderless proteins [33]. Even so, much remains to be learned regarding the role of caspase-1 activation in the control of bacterial infection. In cervical epithelial cells infected by *C. trachomatis*, caspase-1activation contributes to the development of chlamydial infection [81], but caspase-1-dependent caspase-7 activation restricts *Legionella pneumophila* replication in macrophages and in mice [96]. In mycobacterial infection, overexpression of caspase-1 represses *M. tuberculosis* growth in THP-1 macrophages [39]. In this study, inhibition of caspase-1 activity by Z-YVAD-FMK resulted in higher bacterial growth, suggesting that caspase-1 activity is required for restriction of *M. kansasii* growth in macrophages. Secretion of IL-1β is downstream from caspase-1 activation, and IL-1 has been implicated in controlling a variety of intracellular pathogens such as *Listeria*, *Leishmania*, and *M. tuberculosis* [50], [51], [97]–[100]. Our result that blocking IL-1β with IL-1β specific neutralizing antibody led to significantly higher levels of intracellular *M. kansasii*, and that treatment of infected cells with IL-1β reduced the bacterial load, suggested that caspase-1-dependent IL-1β secretion is critical for control of *M. kansasii* infection. The secreted IL-1β might be sensed by IL-1R. Possibly, IL-1R signaling promotes phagolysosomal maturation, which enhances bacterial degradation and clearance, as reported by Master et al. [38]. Concordantly, a previous study with IL-1R1 knockout mice demonstrated that blocking of IL-1-mediated signaling reduced the ability to clear *M. kansasii* from the lungs of IL-1R1 deficient mice [48]. These results highlighted the important role of IL-1βand IL-1R signaling pathway in defence against *M. kansasii* infection, and provided another evidence for the protective role of IL-1β in mycobacterial infection.

IL-1β signaling has been reported to play an important role in the control of mycobacterial infection and granuloma formation [38], [39], [47]–[49], [99]–[103]. In humans, IL-1β is upregulated at the site of mycobacterial infection, and genetic studies demonstrated an association of polymorphisms in the *IL-1* or *IL-1R* genes with tuberculosis susceptibility and disease expression [104]–[107]. Notably, higher levels of IL-1β and NLRP3 mRNA were observed in monocyte-derived macrophage from active tuberculosis patients as compared with healthy subjects [39], suggesting the involvement of NLRP3 inflammasome in human response to mycobacterial infection. Consistent with our results, the involvement of NLRP3/ASC in controlling mycobacterial infection *in vitro* has been reported [41]. However, recent studies using NLRP3-, ASC-, or caspase-1-deficient mice demonstrated that NLRP3/ASC inflammasome is not essential for the control of *M. tuberculosis* infection *in vivo* [108]–[110], although it cannot be excluded that potential compensatory mechanisms can overcome NLRP3/ASC inflammasome dependence in these deficient mice [110]. Thus, the role of NLRP3 inflammasome for antimycobacterial response seems to be controversial. A recent study demonstrated that NLRP3 inflammasome activation is disparate between human and mouse during *Francisella* infection [111], [112], indicating that the human innate response to intracellular pathogens may be distinct from the murine response. Accordingly, the exact role of NLRP3 inflammasome for the control of mycobacterial infection should be carefully evaluated. Furthermore, the protective role of NLRP3 inflammasome against *M. kansasii* infection *in vivo* will be clarified in future studies.

**Figure 6:**
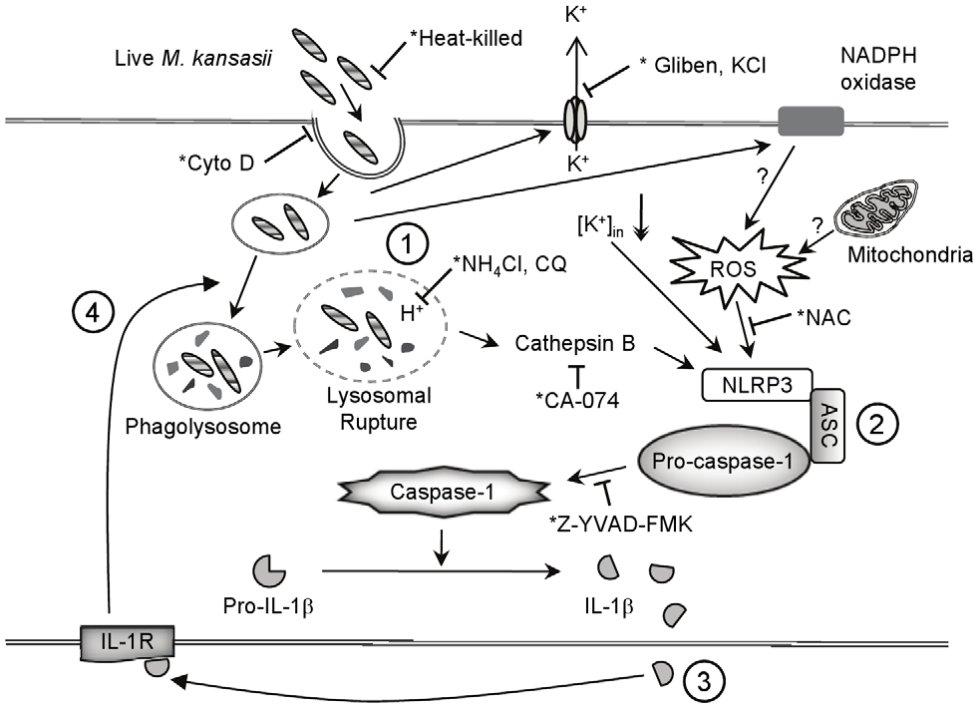
NLRP3/ASC inflammasome activation restricts *Mycobacterium kansasii* infection. During *M. kansasii* infection, live and intracellular bacteria trigger potassium efflux, ROS production and lysosomal damage with cathepsin B release (1), and then lead to NLRP3/ASC inflammasome activation, resulting caspase-1 activation and IL-1βsecretion (2). IL-1β derived from inflammasome activation is released and followed by the engagement of its receptor (IL-1R) (3). IL-1R signaling promotes phagosome maturation that ultimately leads to phagolysosome fusion and bacterial degradation (4).

In conclusion, this report demonstrates that the NLRP3 inflammasome was activated by live intracellular *M. kansasii* through a process involving low intracellular potassium concentration, higher ROS, and active cathepsin B. As a consequence, activated caspase-1 by inflammasome activation triggers the processing and release of IL-1β,which is required for macrophage immunity against *M. kansasii* infection (Figure 6).

## Materials and Methods

### Cells, Bacteria, and Chemical Reagents

THP-1 cells, a human acute monocytic leukemia cell line, were obtained from American Type Culture Collection (ATCC) and were cultured in RPMI 1640 complete medium (Invitrogen) with 10% heat-inactivated fetal bovine serum (HyClone) and 1× antibiotic-antimycotic (Invitrogen) at 37°C with 5% CO_2_ in a humidified incubator. THP-1 stably expressing shRNA against NLRP3, ASC, and nontarget control were obtained as previously described [113], [114]. The *M. kansasii* strain (ATCC12478) was obtained from ATCC and grown at 35°C on Middlebrook 7H11 agar medium (Difco Laboratories) supplemented with 10% OADC (oleic acid, albumin, dextrose, catalase; Becton Dickinson). Heat-killed bacteria were prepared by incubation for 30 min at 80°C, and loss of viability was confirmed by plating on 7H11 plates [115]. Phorbol 12-myristate 13-acetate (PMA), glibenclamide, bafilomycin A1, and Z-YVAD-FMK were purchased from Enzo Life Sciences. LPS (*Escherichia coli* serotype O111:B4), nigericin, N-acetyl-L-cysteine (NAC), cytochalasin D, chloroquine diphosphate, potassium chloride (KCl), ammonium chloride (NH_4_Cl) and dimethyl sulfoxide (DMSO) were from Sigma-Aldrich. The cathepsin B inhibitor, CA-074-Me, was from Calbiochem. The cytotoxicity of reagents used for inhibition studies were evaluated by using the CytoTox 96 Non-Radioactive Cytotoxicity Assay according to the manufacturer's instructions (Promega) (Figure S2).

### Bacterial infection of macrophages

THP-1 cells were differentiated into adherent macrophages by overnight culture in complete medium supplemented with 500 ng/ml PMA, and allowed to rest for 2 days prior to infection. THP-1 derived macrophages were challenged with bacterial suspensions prepared in supplemented medium without antibiotics at the indicated multiplicity of infection (MOI). When using inhibitors or other reagents, cells were preincubated 60 min with inhibitors or other reagents at the indicated concentrations before bacterial infection.

### Cytokine measurement by enzyme-linked immunosorbent assay (ELISA)

To determine IL-1β levels in supernatants from *M. kansasii*-infected cells, the DuoSet ELISA development system kit (R&D Systems) for human IL-1β was used according to the manufacturer's directions. ELISA plates were analyzed using an Emax Microplate Reader (Molecular Devices) at 450 nm.

### Western blotting

Cell culture supernatants from infected macrophages were resolved on 12% SDS-polyacrylamide gels and electrotransferred onto the polyvinylidene difluoride membranes (Millipore). For detection of the active caspase-1 subunit (p20), the membranes were probed with 1∶1000 diluted rabbit anti-human caspase-1 antibody (Millipore) and 1∶10000 diluted horseradish peroxidase-conjugated anti-rabbit IgG antibodies (Santa Cruz Biotechnology). For detection of pro-IL1β and mature IL-1β (p17), the blot was probed with 1∶1000 rabbit anti-human IL-1β antibody (Santa Cruz Biotechnology) and cleaved IL-1β antibody (Cell Signaling), respectively. To detect NLRP3 and ASC, the blots were probed with 1∶1000 rabbit anti-human NLRP3 antibody (Sigma) and mouse anti-human ASC antibody (Santa Cruz Biotechnology), respectively. The signals on the blots were visualized using the enhanced chemiluminescence system (Millipore).

### RNA isolation and PCR

Total RNA was isolated using the Total RNA Mini Kit (Geneaid), reverse transcribed into cDNA (Superscript III, Invitrogen) and analyzed for NLRP3 and ASC mRNA expression by RT-PCR using the following primer pairs. The primers for human GAPDH were 5′-AACGGATTTGGTCGTATTGGGC-3′ forward and 5′-CTTGACGGTGCCATGGAATTTG-3′ reverse. Primers for human NLRP3 were 5′-CTTCTCTGATGAGGCCCAAG-3′ forward and 5′-GCAGCAAACTGGAAAGGAAG-3′ reverse. Primers for human ASC were 5′-ATCCAGGCCCCTCCTCAGT-3′ forward and 5′-GTTTGTGACCCTCGCGATAAG-3′ reverse.

### Evaluation of intracellular bacterial viability by the Colony Forming Unit assay

THP-1 cells (5×10^5^ cells/well) were added to 24-well plates and differentiated into macrophages with PMA. Monolayers of macrophages were infected by *M. kansasii* at an MOI of 1. After 1 h, the medium was removed and the wells were washed with serum-free medium to remove extracellular bacteria and then fresh medium was added. The infected cells were further incubated at 37°C for the indicated time. Some of the infected cells were treated with caspase-1 inhibitor, IL-1β neutralizing antibody (R&D Systems) or exogenous IL-1β (R&D Systems) for different time intervals as described. To evaluate the intracellular bacterial load, cells were lysed with 500 µl of sterile water with 0.1% triton X-100, and the number of viable intracellular bacteria was counted by plating serial dilutions of the lysis solution onto Middlebrook 7H11 agar plates.

### Statistical analysis

All experiments were performed at least three times, and the results are presented as the mean ± standard deviation (SD). Statistical comparisons were performed using Student's *t* test.

## Supporting Information

Figure S1 **IL-6 production is unimpaired in NLRP3 or ASC knockdown cells.** To determine whether the ability to generate pro-IL-1β in response to LPS is diminished in NLRP3 or ASC knockdown cells. ASC, NLRP3, or nontarget control (sh Ctrl) knockdown cells were treated with 1 µg/ml LPS or *M. kansasii* at an MOI of 10. IL-6 in supernatant was measured by ELISA (R&D Systems). Values represent the mean ± standard deviations of at least three independent experiments. These results indicated that ASC and NLRP3 knockdown cells can produce IL-6 normally in response to LPS or *M. kansasii*.
(TIF)

Figure S2 **No apparent cytotoxic effects of inhibitors on THP-1 cells in the experimental conditions.** To evaluate cytotoxic effects of inhibitors used in this study, THP-1 derived macrophages were treated with the indicated pharmacological inhibitors. Cytotoxicity was quantitated by measurement of lactate dehydrogenase (LDH) activity in the culture supernatants using a CytoTox 96 assay kit (Promega) according to the manufacturer's protocol. Error bars represent standard deviation of at least three independent experiments. These results indicated that the experimental treatments have no apparent cytotoxic effects.
(TIF)
