# Supplementary figures and images for: Activation of an NLRP3 Inflammasome Restricts Mycobacterium kansasii Infection
Source: PLoS One. 2012 Apr 30;7(4):e36292. doi: 10.1371/journal.pone.0036292 (PMC3340363; doi:10.1371/journal.pone.0036292)

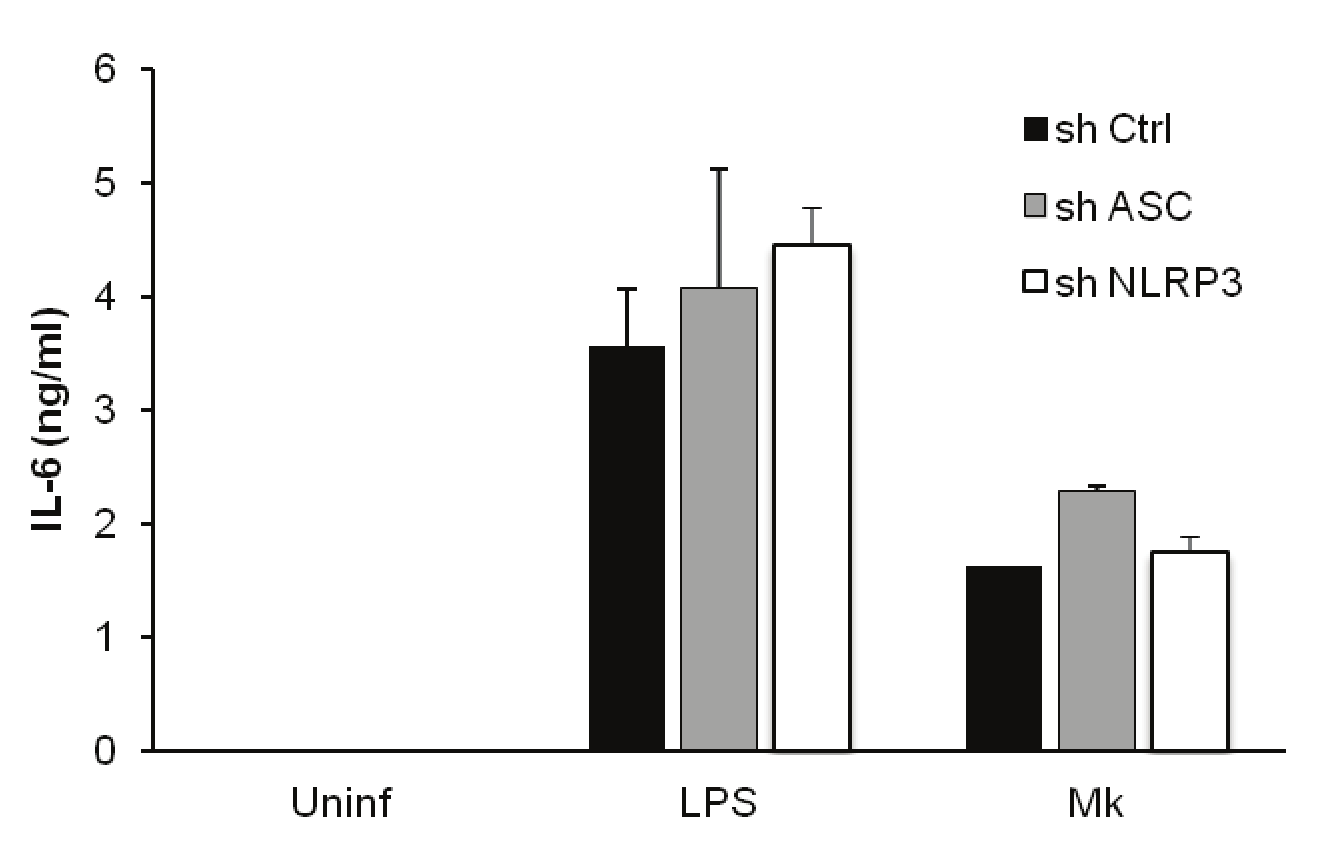

Supplement: Figure S1 — IL-6 production is unimpaired in NLRP3 or ASC knockdown cells. To determine whether the ability to generate pro-IL-1β in response to LPS is diminished in NLRP3 or ASC knockdown cells. ASC, NLRP3, or nontarget control (sh Ctrl) knockdown cells were treated with 1 µg/ml LPS or M. kansasii at an MOI of 10. IL-6 in supernatant was measured by ELISA (R&D Systems). Values represent the mean ± standard deviations of at least three independent experiments. These results indicated that ASC and NLRP3 knockdown cells can produce IL-6 normally in response to LPS or M. kansasii. (TIF) [file pone.0036292.s001.tif]

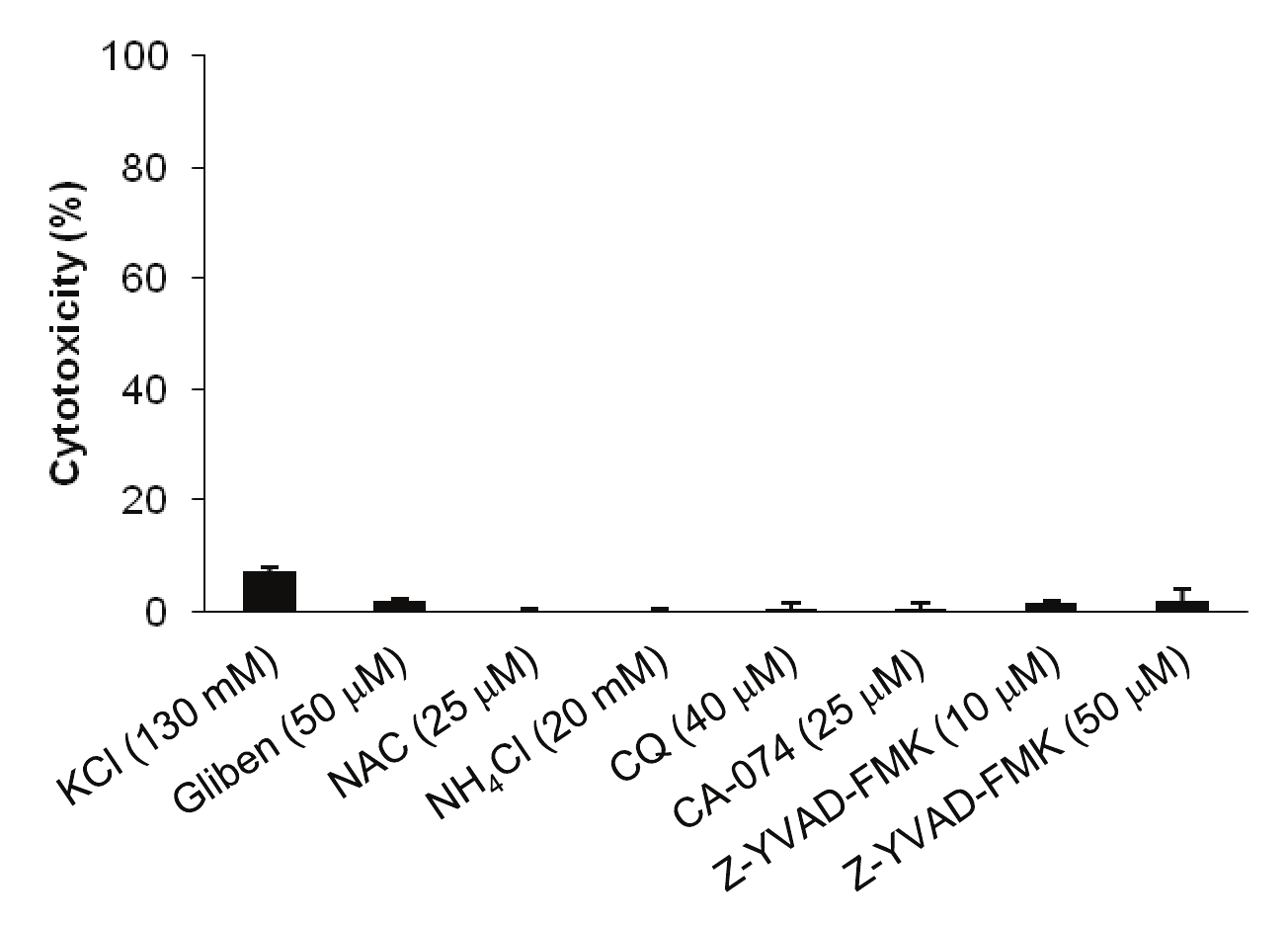

Supplement: Figure S2 — No apparent cytotoxic effects of inhibitors on THP-1 cells in the experimental conditions. To evaluate cytotoxic effects of inhibitors used in this study, THP-1 derived macrophages were treated with the indicated pharmacological inhibitors. Cytotoxicity was quantitated by measurement of lactate dehydrogenase (LDH) activity in the culture supernatants using a CytoTox 96 assay kit (Promega) according to the manufacturer's protocol. Error bars represent standard deviation of at least three independent experiments. These results indicated that the experimental treatments have no apparent cytotoxic effects. (TIF) [file pone.0036292.s002.tif]
